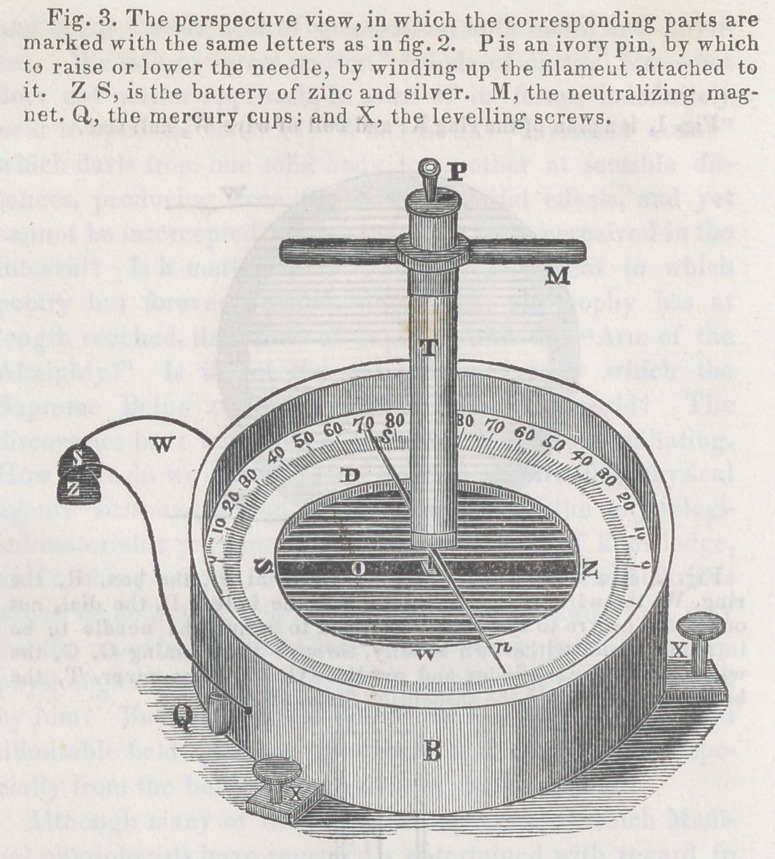# An Account of the Newly Invented Galvanometer

**Published:** 1834

**Authors:** John Locke

**Affiliations:** Cincinnati


### Art. III.—Dr. Locke's Galvanometer.

*An Account of a New Galvanometer, invented by* John Locke, M. D., *of Cincinnati, in a Letter to the* Editor.

*Dear Sir,*—The account of the instrument which I have had the good fortune to bring to a surprising degree of delicacy, as it appeared in the two last numbers of Silliman's Journal, was somewhat defective, in consequence of the progressive manner in which my discoveries were made and communicated. This circumstance, together with the general attention which your journal receives in the west, render it proper that I should comply with your flattering request, that I should make a communication to you on the subject.

In the winter of 1832 and 3, the duty devolved upon me to deliver a course of instructions in Chymistry to the first class of the Mechanics' Institute. In qualifying myself with the proper knowledge of the new science of Electro-Magnetism, I found myself unable, from the works within my reach, to get an *intelligible* account of the Galvanometer, or Electro-Magnetic Multiplier. I finally undertook to supply the deficiency by inventing one, dependent upon the principles established by Oerstead. Compelled thus to operate originally, I have probably succeeded better than I should have done with more assistance. The essential principle of the instrument was communicated to Prof. Silliman, in March, of 1833, but the arrangement of the subordinate parts was not completed until December of the same year, when an account of it was published in the Cincinnati Chronicle. A description nearly complete, was published in Silliman's Journal, of April, and an additional account of its *thermo*-electrical delicacy, in that of July of the present year. We shall give an abstract of the whole below, after having given a short description of the original Galvanometer or Electro-Magnetic Multiplier of Schweigger. This, which is the common Galvanometer, consists essentially of from 40 to 100 feet of copper wire, bound with sewing silk, as bonnet wire is with cotton, and coiled from 40 to 100 times round in the manner of a short skein or hank of thread. This skein of silk-bound wire is bent into an oblong form, like a long link of a chain, say 2 by 5 inches. This being placed edgewise, and a compass needle freely suspended in its centre by a pivot, or by a filament of silk, is ready for use. Let it be placed in the meridian, so that the needle will lie in the same direction as the wires, and pass a current of galvanic electricity through the wire, by connecting one of the projecting ends with the positive, and the other with the negative pole of even an exceedingly small battery, and the needle will be deflected partially or entirely, *at right angles to the wire.* Reverse the current of electricity, and the motion of the needle will be reversed. If a piece of zinc, as large as a five cent piece of silver, be soldered to one end of the wire, this, with the other end of the wire, will form a battery, which, by the application of a weak acid, will deflect the needle perhaps 45 degrees. As this instrument indicates the direction of the galvanic current, and measures its intensity, its name is appropriate. It is remarkable, that although so sensible to *galvanic* currents, it is ordinarily not affected in the least by electricity from the common electrical machine. For a more detailed account of this instrument, let me refer the reader to a work on electro Magnetism, by Jacob Green, M. D., Professor of Chymistry in Jefferson Medical College, Philadelphia; a work which I cheerfully recommend to all who wish to peruse the principles of the science in a condensed form.

Having given this sketch for the satisfaction of those who may not have access to a better, I proceed to a description of the proper subject of this paper.

### *Description of Locke's Discoid Galvanometer.*

The essential part of this instrument, instead of being a hank or heaped up coil, is spread out, and wound over the flat sides of a ring of boxwood.

The outside diameter of the ring is 3¾ inches, and the inside diameter 3 inches. Its thickness is ¼ of an inch. The outside edges of it are cut or notched, like the teeth of a clock wheel, in order to cause it to hold the spiral wire which is wound around in a single layer, the several parallel turns being as close as possible without being in contact, except at the diameter of the ring where an opening of a tenth of an inch is left for the introduction of a magnetized needle within the coil. This flat coil is put into the bottom of a cylindrical box, turned in the shape of a snuff box, four and a half inches in diameter and one and a half deep, outside dimensions; the two projecting ends of the wire being passed through near the bottom of it. Immediately above this coil is the card or divided circle, which is attached to a thin wooden ring. The needle is suspended by a *single filament of the silk worm.* It is made of a simple steel wire, *partly* counteracted in its tendency to settle in the meridian, by being connected with a second and more delicate reversed needle, made of the smallest species of main-spring. This second needle is parallel to the principal one, three-eighths of an inch above it, and with its poles reversed in the manner of M. Nobili. It is intended to act chiefly as an INDEX, and swings above the card. The two needles are firmly connected by a brass wire. The whole is closed by a glass cover, in the manner of a common compass, which it nearly resembles in external appearance. The glass is, however, pierced in its centre by a hole half an inch in diameter for the insertion of a brass tube four inches high. This tube includes the suspension filament which passes through its top and winds on an ivory peg. The projecting wires ascend in an arch, approach within a quarter of an inch, and terminate, the one in a five cent piece of silver, and the other in a corresponding disk of zinc. Where the wires emerge from the box, they are soldered each to a thimble containing mercury for the purpose of forming a metallic pinion with any other apparatus whatever. A magnet, adjustable by a tubular socket, is fixed upon the upper part of the brass tube. The object of this is to render the needle quite "astatic," which had been rendered partially so by the inverted index needle. This is done by sliding this magnet downward on the tube, with its poles repellent until the needle is just ready to obey its influence and become inverted. I consider it delicately adjusted when a strongly magnetised needle 2½ inches long, weighing 20 grains, will make but three oscillations per minute. It is ascertained by calculation, that the force necessary to deflect such a needle one degree, would be only THE ONE HUNDRED THOUSANDTH PART OF A GRAIN.

*Experiment* 1st. When thus adjusted, *one application of a wine glass of pure water to the small disks, will generate electricity enough to turn the needle once around.*

2d. But it is in Thermo-Electricity* that this instrument probably surpasses all others as yet in common use. If two half inch disks, the one of copper and the other of bismuth, each connected with the mercury cups, or poles of the Galvanometer, be laid in contact, and the end of the finger be applied to one of them, the animal heat will, by passing from one to the other, develope electricity enough through the whole circuit of the Galvanometer wire, to turn the needle several times round.

3d. If one disk be warmed, ten degrees only, above the temperature of the other, and applied to it, the needle will be turned quite around.

4th. A few filings of bismuth being placed between two small plates of copper connected with the mercury cups, and the finger applied to one of them, the needle was deflected 22 degrees.

It had been ascertained, that electricity had been developed not only by two contiguous portions of different metals, but by two parts of the same metal, unequally heated. In order to form a scale of the different powers of the several simple metals, when two parts of the same kind were used, I instituted a course of experiments last April, in which I used hand heat only, the temperature of the air being 56 deg. F.

* Electricity developed by the passage of heat from one piece of metal to another, either in apparent contact, or when soldered together.

The following table as published in Silliman's Journal, for July, contains the results:

I. *Positive metals* in which the current of electricity from one portion of metal to the other proceeds from the heated portion in *coincidence* with the caloric. Deviation of the needle in 4 seconds,

| | By hand heat, from 56° to 95° | By boiling heat, from 56° to 212° |
|---|---|---|
| Antimony, | +8 degrees? | +32 deg.? |
| Silver, | 4 | 16 |
| Copper, | 2 | 8 |
| Gold, | 1½ | 6 |
| Lead, | 0 | 0 |

My experiment on antimony is questionable as to the *degree*, because I could not fashion this refractory metal into the desired form. Lead is sometimes null, and sometimes variable.

II. *Negative Metals*, in which the current of electricity from one portion of metal to the other, proceeds from the colder in *opposition* to the caloric. Deviation of the needle in 4 seconds,

| | By hand heat, from 56° to 95° | By boiling heat, from 56° to 212° |
|---|---|---|
| Bismuth, | 11 degrees. | 44 deg. |
| Iron, | 4 | 16 |
| Zinc, | 1 | 4 |
| Tin, | Imperceptible. | 0½ |
| Lead, | 0 | 0 |

I have thus stated a few experiments, merely to show the degree of delicacy obtained by the instrument. I shall now, perhaps, be asked to specify its peculiar advantages. It is compact and elegant in its form; being entirely enclosed, it is not disturbed by the motions of the air; the suspension is such as to render the friction or rather "tortion" insensible; and the "MULTIPLYING" WIRE BEING THE NEAREST POSSIBLE TO THE NEEDLE, MUST AFFECT IT THE MOST SENSIBLY.

The whole doctrine of Electro and Thermo Magnetism, in general, and the delicate indications of the Galvanometer in particular, overturning many of the acknowledged laws of physiology, and pointing out a new relation and agency of things, is calculated to excite admiration and astonishment, and opens a wide field of speculation, both moral and physical. We talk of "gross matter," "clods of earth," &c.—but does not matter approach, in some of its forms, indefinitely near to spiritual essence? What is this "INVISIBLE AGENT" which darts from one solid body to another at sensible distances, producing even the most powerful effects, and yet cannot be intercepted, apprehended, or even perceived in the interval? Is it matter or is it spirit? Is it that to which poetry has forever pointed, and which philosophy has at length reached, the "Bolt of Jupiter" and the "Arm of the Almighty?" Is it not the universal agent by which the Supreme Being operates upon the material world? The discoveries have been great, and yet they are humiliating. How little do we know! How many more "invisible physical agents" surround us and act upon us? Shall the physiological materialist presume upon his narrow field of knowledge, as if nothing existed beyond his ken? May not the doctrines which he denies, be true, even upon his own plan? and may not the repeated violence done to conscience, produce misery physiologically, through invisible agents not yet discovered by him? But I feel myself admonished to retreat from such illimitable fields as those of physics and ethicks, and especially from the belligerent regions of their confines.

Although many of the sanguine expectations which Medical physiologists have repeatedly entertained with regard to electrical discoveries, have not been realized; yet the subject does not lose its interest. The fact that galvanic electricity excites the muscles of recently dead animals into spasmodic action, that certain animals have within their organization a natural battery, that the nerves and muscles form a generating circuit, that the functions of certain organs have been continued by substituting a galvanic battery instead of the brain, the nervous communication with that organ being severed, the strong probability that perception and volition are carried on by electricity; and lastly, that the generation and expenditure of animal heat are constantly attended by electrical changes, still point to further developements, and require that the educated physician should never be unacquainted with the physical sciences.

## Figures and Tables

**Figure f1:**
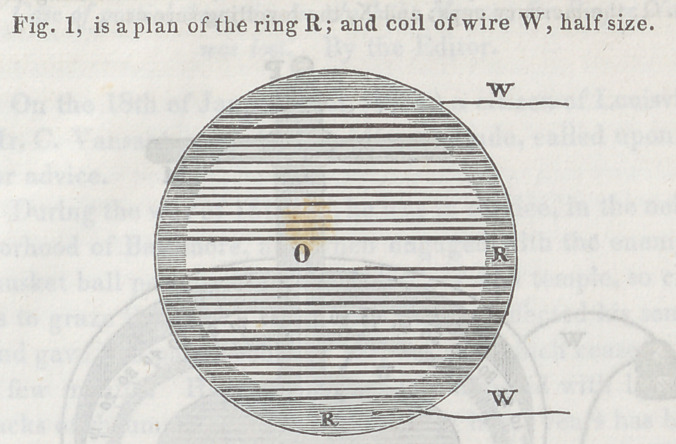


**Figure f2:**
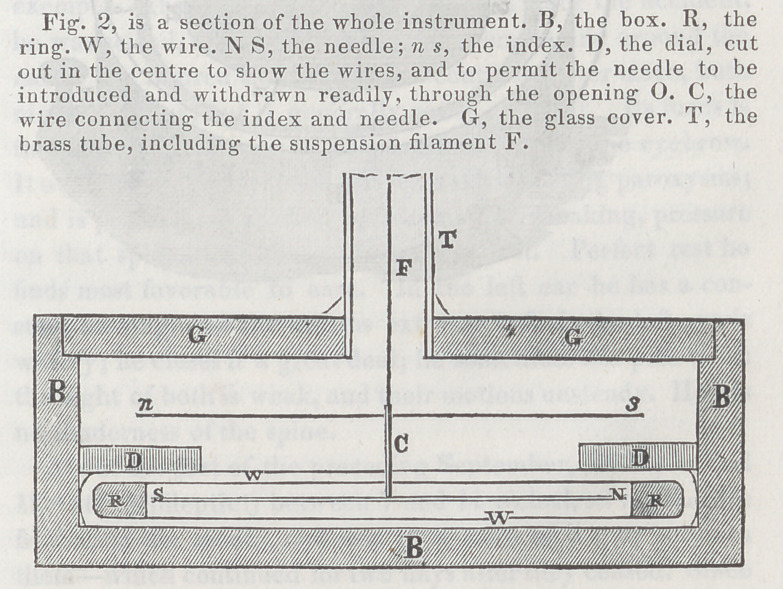


**Figure f3:**